# Study of the Contents of Analogues of Aristolochic Acid in *Houttuynia cordata* by Ultra-High Performance Liquid Chromatography Tandem Mass Spectrometry

**DOI:** 10.3390/foods11030302

**Published:** 2022-01-23

**Authors:** Xuan Yu, Yuan Gao, Ying Xu, Xian Guo, Lan Guo, Ting Tan, Fan Liu, Yiqun Wan

**Affiliations:** 1 College of Chemistry, Nanchang University, Nanchang 330047, China; whalien1214@163.com (X.Y.); a1530953640@163.com (Y.G.); 13517986724@163.com (Y.X.); guoxian1025@163.com (X.G.); guolan@ncu.edu.cn (L.G.); 2 Jiangxi Province Key Laboratory of Modern Analytical Science, Nanchang University, Nanchang 330047, China; tanting@ncu.edu.cn (T.T.); fan_liu@ncu.edu.cn (F.L.)

**Keywords:** *Houttuynia cordata*, aristolochic acid analogues, detection, UHPLC-Q/TOF-MS

## Abstract

In this study, a new and simple method was established for the simultaneous determination of analogues of aristolochic acids (aristolochic acid I, aristolochic acid II, aristolactam I and aristolactam AII) in *Houttuynia cordata* by ultra-high performance liquid chromatography–quadrupole/time-of-flight mass spectrometry (UHPLC–Q/TOF-MS). The samples were ultrasonically extracted with methanol, purified with HC-C18, and then separated on a C18 column (75 × 2.1 mm, 2.0 μm) at 35 °C. Under the optimized conditions, aristolochic acid I (AA-I), aristolochic acid II (AA-II), aristolactam I (AL-I) and aristolactam AII (AL-AII) all showed good linear regression (not less than 0.9987). The average recoveries of the four analytes were within the range of 72.3–105.5%, with the relative standard deviations (RSDs) being ≤7.6%. The proposed method was then applied to the determination of *Houttuynia cordata* samples collected from different regions in China. The results showed that none of the three carcinogenic substances (aristolochic acid I, aristolochic acid II and aristolactam I) were detected in any of the 22 samples collected from 22 different regions of China, while aristolactam AII, which has not been reported to have genotoxicity, was detected in all samples. This study provides a valuable reference for the further safety assessment of *Houttuynia cordata*.

## 1. Introduction

*Houttuynia cordata* is a fresh whole plant or dry ground part of the family Saururaceae [1], which is mainly distributed in the central, southeast and southwest provinces of China, especially in Sichuan, Hubei, Hunan, Jiangsu and other regions. *Houttuynia cordata* can be used as both traditional food and medicine. On the one hand, the young roots and green leaves of *Houttuynia cordata* are the “big road wild vegetable” on public tables in China. In north eastern states of India, it is also often used as a garnish over ethnic dishes and leaf salad [2]. On the other hand, *Houttuynia cordata* has many pharmacological functions, including antiviral [3,4,5], antiallergic [6,7], and anti-inflammatory properties [8,9], and it has also been reported to be used as a bactericidal or antiviral drug in the treatment of some epidemic diseases in China [10] and India [11].

However, a series of “aristolochic acid incidents” have been reported since 1993 [12,13,14]. Aristolochic acids and their metabolite (aristolactam I) have been proved to be nephrotoxic [15,16], carcinogenic [17,18] and mutagenic [19,20]. Moreover, aristolochic acids and plants which contain aristolochic acids were listed as Group 1 human carcinogens by the International Agency for Research on Cancer (IARC) in 2012 [21]. It has been found that *Houttuynia cordata* contains many alkaloids, including aristololactams [22,23], whose structure is similar to that of aristolochic acids and aristolactam I, so rumors that *Houttuynia cordata* is carcinogenic have been spread, causing a panic about its ingestion. Therefore, establishing an accurate, rapid and reliable determination method for aristolochic acids and their analogues to determine whether *Houttuynia cordata* contains carcinogens (aristolochic acids or aristolactam I) is of great significance for the safety assessment of *Houttuynia cordata*.

At present, little research has been conducted on the analysis of aristolochic acid analogues in *Houttuynia cordata*. Chan [24] proposed a LC-MS/MS method for the determination of aristolochic acid I (AA-I) and aristolochic acid II (AA-II) in *Houttuynia cordata*, and found that both of them were not detectable.

In this work, a new and simple analytical method was established for the simultaneous determination of aristolochic acids (aristolochic acid I and aristolochic acid II) and aristololactams (aristolactam I and aristolactam AII) in *Houttuynia cordata* by ultra-high performance liquid chromatography–quadrupole/time-of-flight mass spectrometry (UHPLC–Q/TOF-MS). The proposed method was applied to the determination of *Houttuynia cordata* samples satisfactorily.

## 2. Materials and Methods

### 2.1. Instruments and Regents

**Figure 1:**
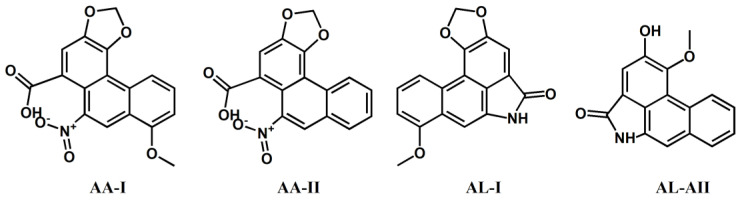
Molecular structures of aristolochic acids and their analogues.

Aristolochic acid I (AA-I, >98%) was obtained from Aladdin Biochemical Technology Co., Ltd. (Shanghai, P.R. China). Aristolochic acid II (AA-II, ≥98%), aristolactam I (AL-I, ≥98%) and aristolactam AII (AL-AII, ≥98%) were obtained from Shanghai Yuanye Bio-technology Co., Ltd. (Shanghai, P.R. China). The chemical structures of the four target compounds (AA-I, AA-II, AL-I and AL-AII) are shown in Figure 1.

Methanol, acetonitrile and ethanol (HPLC grade) were purchased from Tedia (Anhui, China). Formic acid (HPLC grade) was obtained from Anaqua Chemical Supply Inc (Wilmington, DE, USA). Dimethyl sulfoxide (AR) and acetone (AR) were obtained from Sinopharmaceutical Chemical Reagents Co., Ltd. (Shanghai, China). HC-C18 absorbent was purchased from ANPLE Company (Shanghai, China). Silica gel, Kieselguhr G and zeolite were bought from Aladdin Biochemical Technology Co., Ltd. (Shanghai, China), and ultra-pure water was acquired from ELGA LabWater, London, UK.

The UHPLC–MS/MS system was composed of Shimadzu LC-30 UHPLC system (Shimadzu Corporation, Kyoto, Japan) and Triple TOF 5600+ (AB Sciex, Framingham, MA, USA); MS3 mini shaker (Guangzhou Yike Lab Technology LTM Co., Ltd., Guangzhou, China); KQ3200DE type CNC ultrasonic cleaner (Kunshan Ultrasonic Instrument Co., Ltd., Kunshan, China); TDL-5C low-speed table high-capacity centrifuge (Shanghai AnTing Centrifuge, Shanghai, China); TG20.5 high-speed centrifuge (Shanghai Lu Xiangyi Centrifuge, Shanghai, China).

### 2.2. Plant Materials

All the *Houttuynia cordata* samples were collected from 22 different regions of P.R. China and identified by experts from the college of Life Science of Nanchang University.

### 2.3. Preparation of Standard Solutions

Stock standard solutions of AA-I, AA-II and AL-AII were prepared at 500 mg/L with methanol separately. AL-I was prepared at 1000 mg/L with dimethyl sulfoxide. The stock solutions were kept at −20 °C for further experiments. Mixed working standard solutions with different concentrations were prepared by diluting the stock solution using methanol as solvent. All working standard solutions were stored at 4 °C prior to further analysis.

### 2.4. Sample Preparation

The *Houttuynia cordata* samples were firstly dried at 50 °C for 12 h and then crushed to pass through a 80-mesh sieve. A total of 0.20 g of ground *Houttuynia cordata* powder was weighted in a 50 mL tube, and 15 mL methanol was used to extract the sample. After vortexing it vigorously for 2 min, the mixture was ultrasonically extracted at room temperature for 20 min and centrifuged at 3000 rpm for 5 min. A total of 1.6 mL of the supernatant was diluted to 2.0 mL with water. Subsequently, 120 mg of HC-C18 was added to the above diluent solution, and the mixture was vortexed vigorously for 20 min. After centrifuging it for 10 min at 13,800 r/min, the supernatant was collected and dried with nitrogen. Finally, 200 μL of methanol was used to re-dissolve the residue, and the solution was filtered with a 0.22 μm organic filter for UHPLC–Q/TOF-MS analysis.

### 2.5. Chromatographic Conditions

The analysis was performed on UHPLC–Q/TOF-MS using a C18 column (Shim-pack GIST, 75 × 2.1 mm, 2.0 μm) at 35 °C. As the mobile phase, 0.1% (*v*/*v*) formic acid aqueous solution (solvent A) and 0.1% (*v*/*v*) formic acid in acetonitrile were used. The gradient elution program is shown in Table 1. A total of 5 μL of solution was injected into the system, and the solvent flow rate was 0.30 mL/min.

### 2.6. Mass Spectrometry Condition

The target analytes were detected by a Triple TOF 5600+ system which equipped an electrospray ionization (ESI) source on negative mode. The product ion spectra of each target analyte were obtained under high sensitivity mode, and the accumulation time was 100 ms. GAS 1 (ion source gas) was 50 psi; GAS 2 (heater gas) was 50 psi; GAS 3 (curtain gas) was 35 psi; ion source temperature was set as 550 °C, and the ion spray voltage (ISVF) was 5500 V. Each transition was optimized by undertaking multiple injections with differing declustering potential (DP) and collision energy (CE) parameters, respectively. The most abundant and stable product ion was used for quantification, and the second most intense fragment of the product ion was used for qualification (Figure S1). The data was acquired by Analyst software (AB SCIEX, Framingham, MA, USA) and processed by PeakView software (AB SCIEX, Framingham, MA, USA). The mass spectrometric parameters for the target compounds are given in Table 2.

## 3. Results

### 3.1. UHPLC–Q-TOF-MS Conditions

In order to efficiently separate the targets in a shorter time, the chromatographic conditions were optimized. Three mobile phase systems, namely methanol aqueous solution (containing 0.1% (*v*/*v*) formic acid), methanol aqueous solution (containing 0.2% (*v*/*v*) formic acid) and acetonitrile aqueous solution (containing 0.1% (*v*/*v*) formic acid), were investigated. The best separation efficiency and peak shapes were obtained when acetonitrile aqueous solution (containing 0.1% (*v*/*v*) formic acid) was used as the mobile phase. Although AL-I and AA-II were eluted in the same time, the *m*/*z* of the two compounds were different (292.0621 for AL-I and 310.0356 for AA-II). Furthermore, the fragment ions were also different for the two substances (Figure S1). As a result, we can identify the two compounds by MSMS spectrum, and carry out the quantitative analysis of them through the selected product ions [25,26]. Therefore, in the end, the chromatographic separation was carried out using a C18 column with a gradient elution program (Table 1) at a flow rate of 0.3 mL/min, and the temperature of the column was set at 35 °C, with acetonitrile aqueous solution (containing 0.1% (*v*/*v*) formic acid) used as the mobile phase.

The ionization efficiency of the target compound affects the sensitivity of mass spectrometry. In this work, selective ion scanning of the target analytes was performed under the ESI positive and ESI negative modes, respectively. The experimental results showed that under the negative mode, the ionization efficiency and response intensity of the four target compounds were higher than those under the positive mode, so the negative mode was chosen in order to obtain high sensitivity. The influence of other MS conditions, such as DP and CE, were also been investigated. The optimized mass spectrometric parameters for the target analytes are shown in Table 2. The total ion chromatogram (TIC) of the spiked *Houttuynia cordata* sample, and the extracted ion chromatograms (EICs) of the four compounds, both in the spiked sample and the standard solution, are shown in Figure S2. As shown in Figure S2, under the optimized chromatographic and mass spectrometric conditions, the four target analytes were well separated in less than 10 min.

### 3.2. Optimization of Extraction Procedure

**Figure 2:**
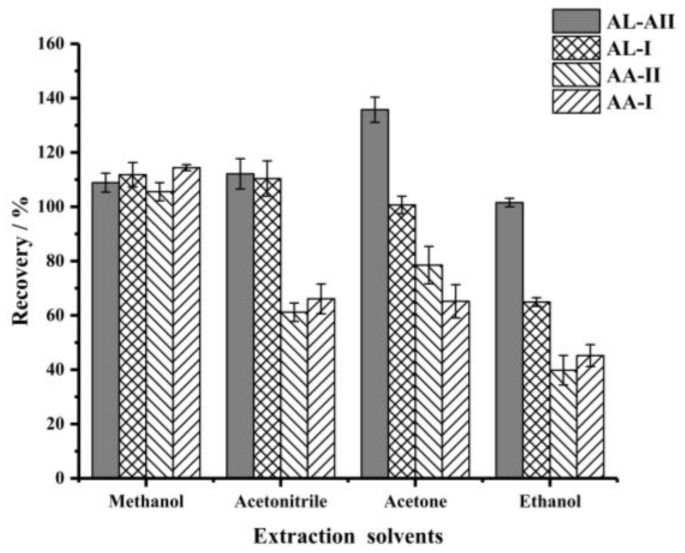
The effect of extraction solvents (volume of extraction solvent, 20 mL; extraction time, 30 min).

**Figure 3:**
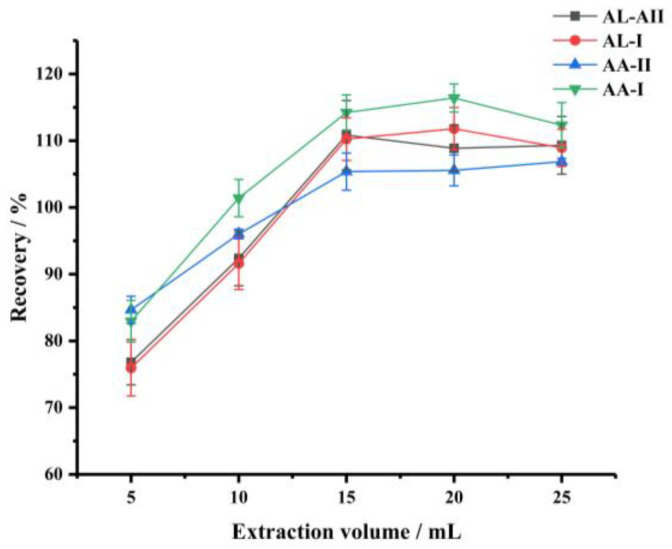
The effect of solvent volume (extraction solvent, methanol; extraction time, 30 min).

To optimize the extraction conditions, the effects of the type and volume of extraction solvent and the extraction time were investigated [27,28]. Firstly, methanol, acetonitrile, acetone and ethanol were used as extraction solvents, respectively [29]. The best extraction recoveries of the four target compounds were achieved by using methanol (Figure 2). Therefore, methanol was selected as the extraction solvent in the following experiments. Next, the effect of solvent volume (5–25 mL) on the extraction efficiency was studied. As shown in Figure 3, the recoveries of the four analytes increased when the solvent volume of methanol was increased from 5 mL to 15 mL, and then remained roughly the same when the solvent volume was increased further, thereby indicating that 15 mL methanol could extract the target compounds thoroughly. Therefore, 15 mL of methanol was selected.

**Figure 4:**
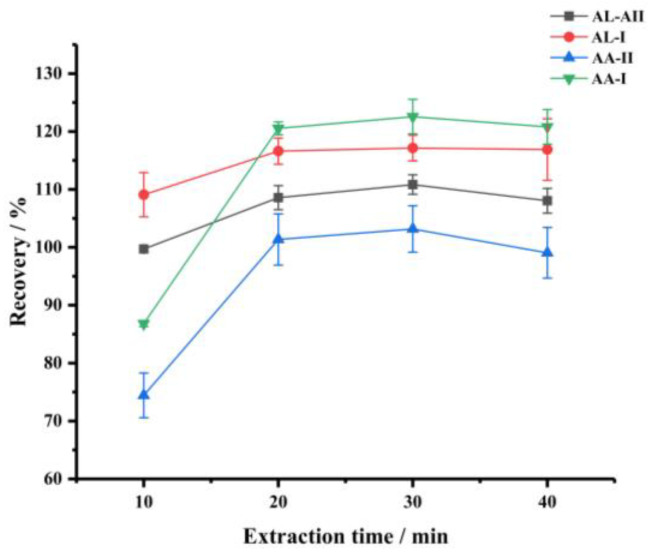
The effect of extraction time (extraction solvent, methanol; volume of extraction solvent, 15 mL).

Moreover, the effect of extraction time was investigated in the range of 10–40 min, and the results are illustrated in Figure 4. As the extraction time increased, the extraction efficiency of the target increased, until a balance was reached after 20 min. Thus, the extraction time was chosen as 20 min in this method.

### 3.3. Optimization of Purification Conditions

Considering the complexity of the *Houttuynia cordata* sample extraction solution (Figure S2C), some compounds may interfere with the analyte detection. Furthermore, the numerous compounds in the solution also can shorten the service life of the chromatographic column. Thus, some solid absorbents were used to remove the interference in the extraction solution in this experiment. The adsorption conditions, including the absorbent type, the methanol concentration in the extraction solution, the amount of absorbents, and the adsorption time, were optimized. Firstly, the purification efficiencies of kieselguhr, silica gel, zeolite and HC-C18 were investigated [30]. The results are shown in Figures S3 and S4. As shown in Figure S3, the recoveries of the four analytes ranged from 90.6% to 100.8% when HC-C18 was used as absorbent, and the recoveries of AL-AII, AL-I and AA-II were higher than when zeolite, silica gel and kieselguhr were used as absorbents. In particular, HC-C18 exhibited the best decoloration effect among the four absorbents (Figure S4) [31]. Thus, HC-C18 was selected as the purification absorbent. Since the high concentration of methanol in the extraction solution could have reduced the purification efficiency of the adsorbent, water was added into the extraction solution to reduce the methanol content. When the methanol content in the extraction solution was decreased to 80% (*v*/*v*), the depigmentation effect was obvious, and the extraction solution became clear after HC-C18 purification (Figure S5). However, when the methanol content was further decreased to 70%, about 20% of AL-AII could be adsorbed by HC-C18. Therefore, the methanol content in the extraction solution was diluted to 80% with water prior to purification by HC-C18. in this experiment.

Then, for the purification of the above 2 mL of extraction solution, the effects of the amount of HC-C18 and the adsorption time were investigated, in the ranges of 40–200 mg and 10~60 min, respectively. The results demonstrated that the best purification efficiency was achieved when more than 120 mg of HC-C18 was used and the adsorption time was more than 20 min. Therefore, the diluted extraction solution was purified with 120 mg of HC-C18 for 20 min in the following experiments.

### 3.4. Linearity, Limits of Detection and Quantification

The linearity of the four analytes was achieved by analyzing a series of concentrations of mixed standard solutions under the optimized experimental conditions. The calibration curves for the four analytes were established by plotting the concentration of standard solutions (x, mg/L) versus peak areas (y). The limits of detection (LOD) and limits of quantification (LOQ) values of the investigated analytes were assessed using the signal-to-noise (S/N) ratios of 3 and 10, respectively. As shown in Table 3, the low correlation coefficients (R^2^) of the calibration curves (≥0.9987) indicated the good linearity of this method. Additionally, this method showed relatively high sensitivity, with the LOQs and LODs of the four compounds being in the range of 2.8 × 10^−3^~0.47 mg/kg and 9.0 × 10^−3^~0.19 mg/kg, respectively.

### 3.5. Recoveries and Precision

In order to further evaluate the accuracy and precision of the method, recovery experiments with three spiked concentration levels were carried out under the optimal conditions. The average recoveries and relative standard deviations (RSD) (*n* = 6) of each compound are presented in Table 4. The average recoveries of the four target compounds (AA-I, AA-II, AL-I and AL-AII) ranged from 72.3 % to 105.5 %, with the RSDs (*n* = 6) being no higher than 7.6 %, indicating the good accuracy, precision and real sample application of the developed method.

### 3.6. Sample Determination

The validated method was then applied to detect aristolochic acids (AA-I and AA-II) and aristolactams (AL-I and AL-AII) in *Houttuynia cordata* samples, which were collected from 22 different regions of China. As shown in Table 5, AL-AII was detected in all the samples, while the other three compounds were not detected in any of the *Houttuynia cordata* samples. In addition, there were also big differences in the content of AL-AII in the *Houttuynia cordata* samples from different regions, with the concentrations ranging from 0.67 mg/kg to 17.24 mg/kg; the highest content was nearly 26 times higher than the lowest content, which may have been due to the different growing conditions, such as altitude, soil, water quality, climate, etc.

## 4. Conclusions

In this study, a new method was developed for the simultaneous determination of analogues of aristolochic acids (AA-I, AA-II, AL-I and AL-AII) in *Houttuynia cordata* by UHPLC–Q/TOF-MS. The method was found to display good accuracy, repeatability and precision. It was applied to the analysis of *Houttuynia cordata* samples collected from different regions of China. The experimental results showed that aristolochic acid I, aristolochic acid II and aristolactam I were all not detectable in any of the samples from 22 different regions of China. This work further confirmed that *Houttuynia cordata* does not contain carcinogenic substances (aristolochic acid I, aristolochic acid II and aristolactam I), and it should serve as a valuable reference for the further safety assessment of *Houttuynia cordata*.

## Figures and Tables

**Table 1:** Mobile phase gradient elution condition.

Time (min)	A (%)	B (%)
0	60	40
7	50	50
7.1	10	90
9	10	90
9.1	60	40
12	60	40

**Table 2:** Mass spectrometric parameters of target compounds.

No.	Compounds	Mode	Precursor Ions (m/z)	Product Ions (m/z)	DP ^b^ (V)	CE ^c^ (eV)
1	AA-I	- ^a^	340.047	296.056 266.058 *	80	15
2	AA-II	-	310.036	250.027 236.047 *	80	14
3	AL-I	-	292.062	249.044 277.039 *	85	25
4	AL-AII	-	264.066	221.049 249.044 *	80	21

* The most abundant and stable product ion for the quantification. - ^a^: The negative ion mode. DP ^b^: Declustering potential. CE ^c^: Collision energy.

**Table 3:** Calibration curves, LODs (the limits of detection) and LOQs (the limits of quantification) of investigated compounds.

Compounds	Linear Range (mg/L)	Calibration Curves	R^2^	LOD ^a^ (mg/kg)	LOQ ^b^ (mg/kg)
AA-I	0.08–2	y = 3288.1x + 247.99	0.9994	0.19	0.47
AA-II	0.04–2	y = 4919.3x + 93.775	0.9997	0.09	0.28
AL-I	0.04–2	y = 15946x−183.82	0.9987	0.09	0.38
AL-AII	0.004–2	y = 3.0 × 10^6^x + 5714.9	0.9999	9.0 × 10^−4^	2.8 × 10^−3^

LOD ^a^: the limits of detection. LOQ ^b^: the limits of quantification.

**Table 4:** Recovery and precision for the determination of the investigated compounds (*n* = 6).

Sample	Compounds	Original (mg/kg)	Added (mg/kg)	Average Recovery (%)	RSD (%)
*Houttuynia cordata*	AA-I	^a^ ND	1.50	87.1	6.2
			4.70	105.5	3.1
			9.40	98.4	3.0
	AA-II	ND	1.50	72.3	2.4
			4.70	101.4	4.3
			9.40	92.1	4.2
	AL-I	ND	1.50	80.7	4.4
			4.70	99.6	3.4
			9.40	91.9	4.0
	AL-AII	8.8	4.0	81.7	7.6
			8.0	99.3	3.2
			12.0	81.0	3.7

^a^ ND: not detected or lower than LOD.

**Table 5:** Analytical results of aristolochic acids and their analogues in *Houttuynia cordata* collected from 22 different regions in China (*n* = 3).

Place of Origin	Concentration (mg/kg)			
	AA-I	AA-II	AL-I	AL-AII
Lu’an, Anhui	^a^ ND	ND	ND	1.13 ± 0.01
Wuxi County, Chongqing	ND	ND	ND	3.21 ± 0.11
Yajiang Town, Chongqing	ND	ND	ND	8.16 ± 0.13
Longyan, Fujian	ND	ND	ND	3.93 ± 0.13
Yongtai, Fujian	ND	ND	ND	4.86 ± 0.04
Zhaoqing, Guangdong	ND	ND	ND	0.67 ± 0.02
Qingyuan, Guangdong	ND	ND	ND	2.95 ± 0.08
Yulin, Guangxi	ND	ND	ND	17.24 ± 0.13
Guiyang, Guizhou	ND	ND	ND	4.73 ± 0.03
Changshun County, Guizhou	ND	ND	ND	0.98 ± 0.02
Xinyang, Henan	ND	ND	ND	3.37 ± 0.09
Yichang, Hubei	ND	ND	ND	0.93 ± 0.02
Zhangjiajie, Hunan	ND	ND	ND	5.69 ± 0.07
Chenzhou, Hunan	ND	ND	ND	1.43 ± 0.04
Wanzai County, Jiangxi	ND	ND	ND	4.37 ± 0.11
Ji’an County, Jiangxi	ND	ND	ND	2.10 ± 0.06
Shanggao County, Jiangxi	ND	ND	ND	3.35 ± 0.10
Linyi, Shandong	ND	ND	ND	2.34 ± 0.09
Mianyang, Sichuan	ND	ND	ND	2.68 ± 0.09
Bazhong, Sichuan	ND	ND	ND	1.03 ± 0.01
Dazhou, Sichuan	ND	ND	ND	1.88 ± 0.04
Lishui, Zhejiang	ND	ND	ND	4.20 ± 0.06

^a^ ND: not detected or lower than LOD.

## Data Availability

Not applicable.

## Supplementary Materials

The following supporting information can be downloaded at: https://www.mdpi.com/article/10.3390/foods11030302/s1, Figure S1. The MS/MS spectra of (a) AA-I, (b) AA-II, (c) AL-I and (d) AL-AII, Figure S2. The extracted ion chromatograms (EICs) of the mix standard solution (A), EICs of spiked *Houttuynia cordata* sample extraction solution (B) and the total ion chromatogram (TIC) of spiked *Houttuynia cordata* sample extraction solution (C). Compounds: 1. AL-AII; 2. AL-I; 3. AA-II; 4. AA-I, Figure S3. The effect of different absorbents, Figure S4: The purification effect of different absorbents (a) before purification, (b) HC-C18, (c) zeolite, (d) silica gel, (e) kieselguhr, Figure S5: The purification effect of different methanol concentrations in the extraction solution (absorbent, HC-C18) a: before purification; b: 90%; c: 85%; d: 80%; e: 70%; f: 50%.

## References

[1] Kumar M., Prasad S., Hemalatha S. (2014). A Current Update on the Phytopharmacological Aspects of Houttuynia Cordata Thunb. Pharmacogn. Rev..

[2] Shingnaisui K., Dey T., Manna P., Kalita J. (2018). Therapeutic Potentials of Houttuynia Cordata Thunb. against Inflammation and Oxidative Stress: A Review. J. Ethnopharmacol..

[3] Chen X., Wang Z., Yang Z., Wang J., Xu Y., Tan R., Li E. (2011). Houttuynia Cordata Blocks HSV Infection through Inhibition of NF-ΚB Activation. Antivir. Res..

[4] Cheng D., Sun L., Zou S., Chen J., Mao H., Zhang Y., Liao N., Zhang R. (2019). Antiviral Effects of Houttuynia Cordata Polysaccharide Extract on Murine Norovirus-1 (MNV-1)—A Human Norovirus Surrogate. Molecules.

[5] Chiow K.H., Phoon M.C., Putti T., Tan B.K.H., Chow V.T. (2016). Evaluation of Antiviral Activities of Houttuynia Cordata Thunb. Extract, Quercetin, Quercetrin and Cinanserin on Murine Coronavirus and Dengue Virus Infection. Asian Pac. J. Trop. Med..

[6] Han E.H., Park J.H., Kim J.Y., Jeong H.G. (2009). Houttuynia Cordata Water Extract Suppresses Anaphylactic Reaction and IgE-Mediated Allergic Response by Inhibiting Multiple Steps of FcεRI Signaling in Mast Cells. Food Chem. Toxicol..

[7] Li G.Z., Chai O.H., Lee M.S., Han E.-H., Kim H.T., Song C.H. (2005). Inhibitory Effects of Houttuynia Cordata Water Extracts on Anaphylactic Reaction and Mast Cell Activation. Biol. Pharm. Bull..

[8] Kim D., Park D., Kyung J., Yang Y.-H., Choi E.-K., Lee Y.-B., Kim H.-K., Hwang B.Y., Kim Y.-B. (2012). Anti-Inflammatory Effects of Houttuynia Cordata Supercritical Extract in Carrageenan-Air Pouch Inflammation Model. Lab. Anim. Res..

[9] Li W., Yang F., Zhan H., Liu B., Cai J., Luo Y., Zhou X. (2020). *Houttuynia cordata* Extract Ameliorates Bladder Damage and Improves Bladder Symptoms via Anti-Inflammatory Effect in Rats with Interstitial Cystitis. Evid.-Based Complement. Altern. Med..

[10] Lau K.-M., Lee K.-M., Koon C.-M., Cheung C.S.-F., Lau C.-P., Ho H.-M., Lee M.Y.-H., Au S.W.-N., Cheng C.H.-K., Lau C.B.-S. (2008). Immunomodulatory and Anti-SARS Activities of Houttuynia Cordata. J. Ethnopharmacol..

[11] Laloo D., Hemalatha S. (2011). Ethnomedicinal Plants Used for Diarrhea by Tribals of Meghalaya, Northeast India. Pharmacogn. Rev..

[12] Lord G.M., Tagore R., Cook T., Gower P., Pusey C.D. (1999). Nephropathy Caused by Chinese Herbs in the UK. Lancet.

[13] Maggini V., Menniti-Ippolito F., Firenzuoli F. (2018). Aristolochia, a Nephrotoxic Herb, Still Surfs on the Web, 15 Years Later. Intern. Emerg. Med..

[14] Vanherweghem J.-L., Depierreux M., Tielemans C., Abramowicz D., Dratwa M., Jadoul M., Richard C., Vandervelde D., Verbeelen D., Vanhaelen-Fastre R. (1993). Rapidly Progressive Interstitial Renal Fibrosis in Young Women: Association with Slimming Regimen Including Chinese Herbs. Lancet.

[15] Cheng C.-L., Chen K.-J., Shih P.-H., Lu L.-Y., Hung C.-F., Lin W.-C., Yesong Gu J. (2006). Chronic Renal Failure Rats Are Highly Sensitive to Aristolochic Acids, Which Are Nephrotoxic and Carcinogenic Agents. Cancer Lett..

[16] Wang X., Giusti A., Ny A., de Witte P.A. (2020). Nephrotoxic Effects in Zebrafish after Prolonged Exposure to Aristolochic Acid. Toxins.

[17] Han J., Xian Z., Zhang Y., Liu J., Liang A. (2019). Systematic Overview of Aristolochic Acids: Nephrotoxicity, Carcinogenicity, and Underlying Mechanisms. Front. Pharmacol..

[18] Stiborová M., Frei E., Breuer A., Bieler C.A., Schmeiser H.H. (1999). Aristolactam I a Metabolite of Aristolochic Acid I upon Activation Forms an Adduct Found in DNA of Patients with Chinese Herbs Nephropathy. Exp. Toxicol. Pathol..

[19] Michl J., Ingrouille M.J., Simmonds M.S.J., Heinrich M. (2014). Naturally Occurring Aristolochic Acid Analogues and Their Toxicities. Nat. Prod. Rep..

[20] Ng A.W.T., Poon S.L., Huang M.N., Lim J.Q., Boot A., Yu W., Suzuki Y., Thangaraju S., Ng C.C.Y., Tan P. (2017). Aristolochic Acids and Their Derivatives Are Widely Implicated in Liver Cancers in Taiwan and throughout Asia. Sci. Transl. Med..

[21] Shang X., You C., Li X., Yuan L., Jin M., Zhang X. (2021). Involvement of 5-HT2 Serotonin Receptors in Cognitive Defects Induced by Aristolochic Acid I in Mice. Toxicology.

[22] Jiang Y., Lu Y., Zhang Y.-Y., Chen D.-F. (2014). Anti-Complementary Constituents of *Houttuynia cordata* and Their Targets in Complement Activation Cascade. Nat. Prod. Res..

[23] Pröbstle A., Bauer R. (1992). Aristolactams and a 4,5-Dioxoaporphine Derivative from *Houttuynia cordata*. Planta Med..

[24] Chan C., Pan G., Chan W. (2021). Analysis of Aristolochic Acids in *Houttuynia cordata* by Liquid Chromatography—Tandem Mass Spectrometry. J. Mass. Spectrom..

[25] Liu F., Li B., Yang Y.L., Wan Y.Q. (2018). A simple and reliable ultra-high performance liquid chromatography coupled with tandem mass spectrometry method for simultaneous quantification of tyrosine and its metabolites in human urine. J. Liq. Chromatogr. Relat. Technol..

[26] Morris B.D., Schriner R.B. (2015). Development of an automated column solid-phase extraction cleanup of QuEChERS extracts, using a zirconia-based sorbent, for pesticide residue analyses by LC-MS/MS. J. Agric. Food Chem..

[27] Chen H., Liu J., Cui M., Chen J., Li X., Chen Y. (2018). Simultaneous determination of four amides in Saururus chinensis by matrix solid phase dispersion and high-performance liquid chromatography method. J. Food Drug Anal..

[28] Abbaspour M., Farajzadeh M.A., Sorouraddin S.M., Mohebbi A. (2019). Monitoring of nine pesticides in different cereal flour samples with high performance liquid chromatography-diode array detection. Anal. Methods.

[29] Wu H.Z., Zhou M., Xu J., Wang J.M., Tong J.Y., Sun N.B., Qian M.R. (2022). Determining a wide range of antibiotics and pesticides in poultry feathers using selective accelerated solvent extraction-liquid chromatography-mass spectrometry. Anal. Methods.

[30] Chen H.J., Ji T., Chen J.W., Li X. (2019). Matrix solid-phase dispersion combined with HPLC-DAD for simultaneous determination of nine lignans in Saururus chinensis. J. Chromatogr. Sci..

[31] Xian Y., Wu Y., Dong H., Chen L., Zhang C., Hou X., Zeng X., Bai W., Guo X. (2019). Modified QuEChERS purification and Fe_3_O_4_ nanoparticle decoloration for robust analysis of 14 heterocyclic aromatic amines and acrylamide in coffee products using UHPLC-MS/MS. Food Chem..

