# Supplementary material for: Study of the Contents of Analogues of Aristolochic Acid in Houttuynia cordata by Ultra-High Performance Liquid Chromatography Tandem Mass Spectrometry
Source: Foods. 2022 Jan 23;11(3):302. doi: 10.3390/foods11030302 (PMC8834043; doi:10.3390/foods11030302)
Supplement: Supplementary file 1 [file foods-11-00302-s001.zip › foods-1565286-supplementary.pdf]

Study of the contents of analogues of aristolochic acid in Houttuynia cordata by ultra-high performance liquid chromatography tandem mass spectrometry

Xuan Yu[1], Yuan Gao[1], Ying Xu[1], Xian Guo[1], Lan Guo[1, 2], Ting Tan[2], Fan Liu[2], Yiqun Wan[1, 2, *]

(1. College of Chemistry, Nanchang University, Nanchang, Jiangxi, P. R. China 330047;

2. Jiangxi Province Key Laboratory of Modern Analytical Science, Nanchang University, Nanchang, Jiangxi, P. R. China 330047)

Xuan Yu: Postal address: Changhai building, 999 xuefu Road, Nanchang University, Nanchang, Jiangxi, P. R. China 330047;

Email address: 1443712462@qq.com

***Corresponding author: Prof. Yiqun Wan**

Postal address: Changhai building, 999 xuefu Road, Nanchang University, Nanchang, Jiangxi, P. R. China 330047;

Tel.: +86 791 88321370; Fax: +86 791 88321370;

E-mail address: wanyiqun@ncu.edu.cn

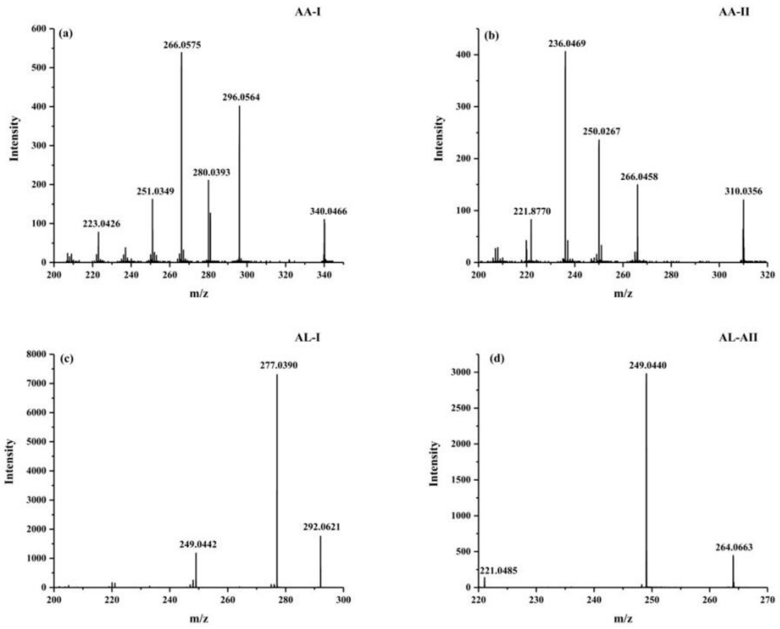

Figure S1. The MS/MS spectra of (a) AA-I, (b) AA-II, (c) AL-I and (d) AL-AII

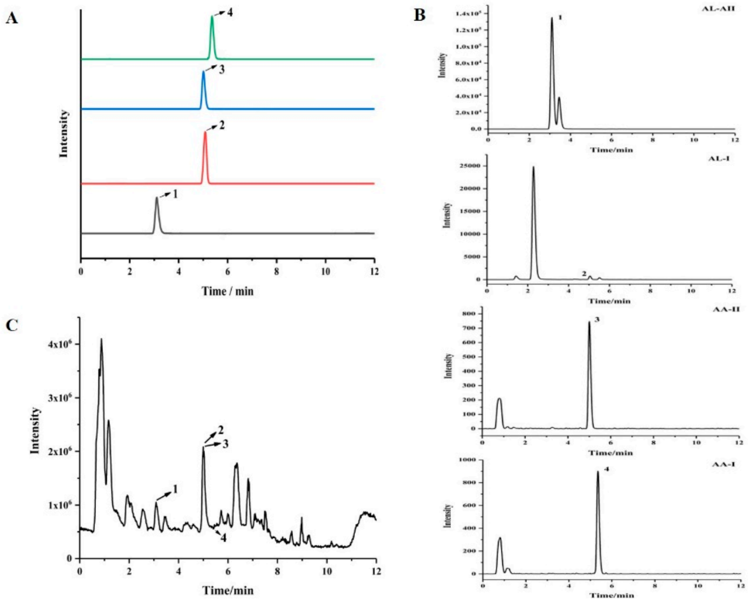

Figure S2. The extracted ion chromatograms (EICs) of the mix standard solution (A) and EICs (B), the total ion chromatogram (TIC) (C) of spiked Houttuynia cordata sample extraction solution. Compounds: 1. AL-AII; 2. AL-I; 3. AA-II; 4. AA-I.

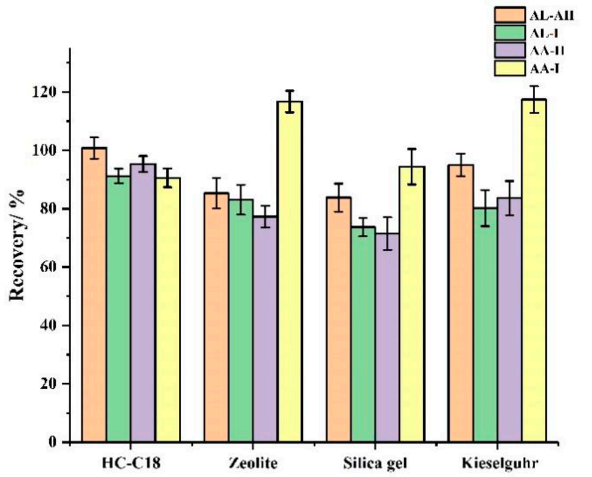

Figure S3. The effect of different absorbents

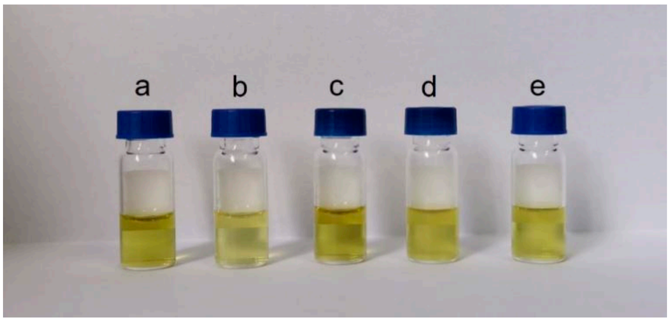

Figure S4. The purification effect of different absorbent

(a) before purification (b) HC-C18, (c) Zeolite, (d)Silica gel, (e) Kieselguhr

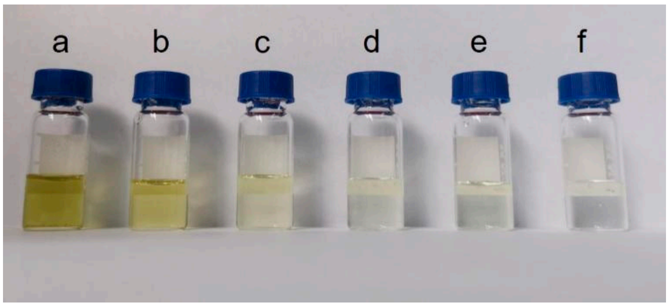

Figure S5. The purification effect of different methanol concentration in the extraction solution (absorbent, HC-C18)

a: before purification; b: 90%, c: 85%, d: 80%, e: 70%, f: 50%
